# Comparative analysis of surgical management approaches for hydatid liver cysts: conventional vs. minimally invasive techniques

**DOI:** 10.1007/s00423-023-03043-8

**Published:** 2023-08-18

**Authors:** Walid Elmoghazy, Jowhara Alqahtani, Seon Woo Kim, Ibnouf Sulieman, Ahmed Elaffandi, Hatem Khalaf

**Affiliations:** 1 Department of Surgery, Organ Transplant Section, Hamad Medical Corporation, P.O. Box 3050, Doha, Qatar; 2 Present Address: Department of Surgery, Sohag University, Sohag, Egypt; 3 Department of Medicine, Weill-Cornell Medical College, Doha, Qatar; 4 Department of Surgery, National Cancer Institute, Cairo University, Cairo, Egypt

**Keywords:** da Vinci robot, Pericystectomy, Hepatectomy, Bile leak, Recurrence

## Abstract

**Introduction:**

Hydatid liver disease is a prevalent condition in endemic areas, particularly in the Middle East and North Africa. The use of laparoscopy as a treatment option has gained popularity. However, there is still ongoing debate regarding the optimal approach for surgical management. In this study, we present our experience with the surgical treatment of hydatid liver disease comparing conventional and minimally invasive approaches, including laparoscopic and robotic options.

**Methods:**

We conducted a retrospective review of patients who underwent surgery for hydatid liver disease at our institution. Data was collected on the patients’ clinical presentations, cyst characteristics, surgical procedures performed, intraoperative findings, and postoperative complications.

**Results:**

A total of 98 hydatid liver cysts were surgically managed in 57 patients. The mean age of the patients was 37.2 ± 10.2 years, with 38 (66.7%) being male. Among the patients, 14 (24.6%) underwent conventional surgery (6 partial pericystectomy, 4 total pericystectomy, and 4 liver resection), 37 (64.9%) underwent laparoscopic surgery (31 partial pericystectomy, 4 total pericystectomy, and 2 liver resection), and 6 (10.5%) underwent robotic surgery (6 partial pericystectomy). There were no significant differences between the conventional surgery and minimally invasive groups in terms of patient age, gender, cyst size, or number. However, laparotomy was associated with a higher number of total pericystectomy and liver resection procedures compared to the minimally invasive approach (*P* = 0.010). Nonetheless, the operation time and blood loss were comparable between both groups. Perioperative complications occurred in 19 (33.3%) patients, with 16 (84%) experiencing minor issues. Bile leak occurred in 8 (14%) patients, resolving spontaneously in 5 patients. There was no significant difference (*P* = 0.314) in the incidence of complications between the two groups. Conventional surgery, however, was associated with a significantly longer hospital stay (*P* = 0.034). During follow-up, there were no cases of mortality or cyst recurrence in our cohort.

**Conclusion:**

Minimally invasive approaches for hydatid liver cysts offer advantages such as shorter hospitalization and potentially quicker recovery, making them valuable treatment options when accompanied by careful patient selection and adherence to proper surgical techniques.

## Introduction

Liver hydatid cyst, also known as echinococcosis, is a condition caused by the *Echinococcus granulosus* tapeworm. This disease is particularly prevalent in rural areas and pastoral communities. It is most commonly observed in various parts of the world, including the Middle East, Africa, South America, and certain regions in Asia such as India, Pakistan, and China [1, 2].

Three primary options are available when treating hydatid liver cysts: chemotherapy, surgery, and percutaneous drainage [3]. Despite that, surgery remains the treatment of choice. The conventional surgical approach to treat hydatid liver cysts typically involves open surgery. This procedure entails making a large incision to access the cysts and remove them manually. Although effective, this technique carries certain drawbacks, such as increased postoperative pain, prolonged hospital stay, and a higher risk of complications, including wound infections and abdominal adhesions [4].

Extensive surgical trauma can also lead to delayed recovery and extended time away from regular activities [3]. The minimally invasive techniques were introduced in 1992 with the first successful laparoscopic pericystectomy, and since then, many reports have been published showing an increased number of laparoscopic hydatid treatments [5]. The general acceptance of laparoscopy was a slow process and was delayed by the concern about possible intraoperative spillage, anaphylactic shock, and cystic rupture due to the pneumoperitoneum. However, pneumoperitoneum, which was deemed to cause cystic rupture, came out to be protective. In addition, it offers several advantages in terms of reduced postoperative pain, shorter hospital stays, quicker recovery times, and improved cosmetic outcomes due to more minor scars [4, 5].

Recently, robotic-assisted surgery, a more advanced form of minimally invasive surgery, has been used for treating hydatid liver cysts, and there are few case reports published showing its feasibility and safety [6–8].

However, a considerable debate remains regarding the optimal surgical management approach. This report aims to analyze cases of surgically managed hydatid liver cysts and compare the outcomes of conventional surgical techniques with minimally invasive approaches, including laparoscopy and robotic surgery options.

## Patient and methods

### Study population

The study population included all patients diagnosed with hydatid liver disease who underwent surgical management at our institution. Patients managed solely by medical treatment or endoscopic approaches were excluded. The study had a minimum follow-up period of 1 month after surgery. Ethical approval for the study was obtained from the institutional review board.

### Diagnosis

The diagnosis of hydatid liver disease relied on imaging modalities such as ultrasonography, computed tomography (CT), and/or magnetic resonance imaging (MRI). These imaging techniques were used to determine the size, location, number of cysts, and any associated complications such as biliary communication or cyst rupture. Serological testing using enzyme-linked immunosorbent assay (ELISA) was routinely performed to detect antibodies specific to *Echinococcus granulosus*

### Data collection

Data were retrospectively collected from a prospectively maintained database, encompassing patients diagnosed with hydatid liver disease and managed through surgery, including cases with biliary communication. Three patients with incomplete records were excluded. The collected data encompassed various aspects, including patient characteristics (age, gender, comorbidities, previous surgical history, body mass index), American Society of Anesthesia classification (ASA) [9], hydatid cyst characteristics (size, number, location, viability, presence of biliary communication), imaging results (ultrasonography, CT, or MRI), operative details (operative time, surgical approach, procedures performed, blood loss, blood transfusion, intraoperative complications), postoperative complications, hospital stay, patient survival, and disease recurrence.

### Operative details

The surgical procedures performed using different approaches were similar and included the following techniques: lagrot partial pericystectomy which involved resection of the corticalized pericyst up to the border with the healthy liver parenchyma. Total pericystectomy that required complete excision of the pericyst after inactivating the cyst content. Anatomical liver resection was performed based on the involved liver segments using a cavitron ultrasonic surgical aspirator.

Choice of scolicidal solution and its volume depended on factors such as cyst size, number, presence of biliary communication, and surgeon’s preference. Hypertonic saline solutions with concentrations ranging from 5 to 20% were used. Hypertonic solution with a 20% concentration was used in cysts with no biliary communication to avoid the risk of sclerosing cholangitis, and in large cysts (>10 cm) as it gets diluted inside the cyst; however, small cysts and those with suspected biliary communication were injected with a lower concentration of the hypertonic saline.

The surgeon’s level of expertise determined the surgical method employed. Additionally, the conventional approach was utilized for cases involving ruptured and recurring liver cysts, as well as cysts that were difficult to access by the minimally invasive techniques.

### Surgical techniques for the conventional group

A right subcostal incision with a midline extension was made. Adhesions between the cysts and surrounding structures were lysed. The peritoneal cavity was isolated using dressings soaked in 20% hypertonic saline (for isolating a limited area) or a hypotonic solution (for isolating large peritoneal areas as in bilobar liver disease to minimize the risk of hypernatremia) to prevent dissemination. The cyst was punctured with a needle to release the cystic aspiration fluid, relieve tension, and allow injection of the scolicidal agent. After 5–20 min, the hydatid content was aspirated. Cystotomy was then performed from the puncture site, followed by extraction of the germinal membrane and daughter vesicles.

### Surgical techniques for the laparoscopy group

The procedure was performed with the patient in the supine position using four ports. A supraumbilical 12 mm port was used to insert a 30° telescope. A 12 mm port was inserted at the mid-clavicular line for cysts in the right lobe or the epigastric area for cysts in the left liver lobe.

Two 5 mm ports were inserted based on the cyst’s location. A pneumoperitoneum (12–15 mm Hg) was established using carbon dioxide, and adhesions between the cysts and neighboring organs were lysed. The cysts were isolated from the peritoneal cavity using dressings soaked with a scolicidal agent (20% hypertonic saline or a hypotonic saline solution). Aspiration of the cyst cavity was performed to relieve tension and create space for the scolicidal agent. After 5–20 min, the cyst content was aspirated, followed by cystostomy and removal of daughter cysts and the membrane using a plastic bag.

### Surgical techniques for the robotic group

The patient was positioned in a split-leg position with the head elevated by approximately 30°. Four ports of 8 mm diameter were used, with the camera lens positioned just below the umbilicus. The assistant port was placed in the right or left mid-clavicular lines below the umbilicus level, depending on the cyst’s location. A pneumoperitoneum (12–15 mm Hg) was established using carbon dioxide, and adhesions between the cysts and neighboring organs were lysed. The cysts were isolated from the peritoneal cavity using dressings soaked with a scolicidal agent (20% hypertonic or a hypotonic saline solution). Aspiration of the cyst cavity was performed to relieve tension and create space for the scolicidal agent. After 5–20 min, the cyst content was aspirated, followed by cystostomy and removal of daughter cysts in a plastic bag.

### Outcome measures

The surgical approaches were compared regarding operative time, blood loss, the need for blood transfusion, intraoperative and postoperative complications, and the hospital stay. The secondary outcome measures included disease recurrence and patient survival. Due to the small number of cases done by the da Vinci robot, they were analyzed together with the laparoscopic cases as the minimally invasive group.

Postoperative complications (within 1 month from surgery) were assessed in detail. Complications were graded according to the Dindo-Clavien classification [10] to assess the severity, and major complications were defined as a grade > II. If a patient had two or more complications, the most severe was considered for grading.

Bleeding is defined as a drop of hemoglobin level > 3 g/dl after surgery compared to postoperative baseline level and/or any postoperative transfusion of packed red blood cell units for falling hemoglobin and/or the need for invasive re-intervention [11].

Bile leakage is defined as bilirubin concentration in the drain fluid at least three times the serum bilirubin concentration on or after postoperative day three or as the need for radiologic or operative intervention resulting from biliary collections or bile peritonitis, according to the International Study Group of Liver Surgery [12].

### Statistical analysis

The collected data were summarized by calculating the mean plus standard deviation (SD) for continuous variables and presenting the frequency (percentage) for categorical data. To compare the conventional surgery group with the minimally invasive group, an independent sample Student’s *t*-test was used for variables that followed a parametric distribution, while Mann–Whitney *U* tests were employed for non-parametric variables. The association between the groups for categorical variables was assessed using either Pearson’s chi-square test or Fisher’s exact test, depending on appropriateness. A significance level of less than 0.05 was considered statistically significant. The statistical analysis was performed using SPSS software version 26 (IBM SPSS Inc., USA).

## Results

Over a period of 10 years, 73 patients were diagnosed with hydatid liver disease. Patients who had inactive cysts or treated medically or by endoscopy and those with incomplete records were excluded. A total of 57 patients with 98 hydatid liver cysts were surgically managed and included in our study. The mean age was 37.2 ± 10.2 years, and 38 (66.7%) were males. The mean body mass index of the patients was 27 ± 4.3 kg/m^2^. The mean follow-up from the surgical intervention was 1.2 ± 1.4 years (range 0.1–8.4 years). Risk factors such as dog exposure were identified in 9 (15.8%) patients only. Most of the patients, 33 (58%), presented with abdominal pain, 6 (10.5%) patients had cholangitis, 6 (10.5%) had abdominal mass, and 12 (21%) patients were incidentally diagnosed with hydatid liver cysts. Two patients were operated upon due to ruptured cysts, and two patients were referred due to recurrent disease after surgery elsewhere.

Upon evaluating the patients based on their geographic region and nationalities, a significant observation emerged regarding the majority of the cohort, which comprised 36.8% of individuals from the Middle East. Within this subgroup, Qatari patients constituted 7 out of the 21 individuals. In contrast, Europeans represented the smallest group of patients admitted for treatment.

Regarding prevalence, Asian and African patients demonstrated an equal representation, comprising 29.8% of the cohort. These findings align with the population demographics of Qatar.

The blood tests at the time of surgery showed serum bilirubin of 20.0 ± 29.1 μmol/L, aspartate aminotransferase (AST) of 58.9 ± 94.8 μmol/L, alanine aminotransferase (ALT) of 88.0 ± 167.6 μmol/L, and alkaline phosphatase (ALP) of 121.7 ± 93.1 μmol/L. 51 (89.5%) patients tested positive for echinococcus antibodies.

Hydatid liver cysts were single in 37 (65%) patients and multiple in 20 (35%). The cyst diameter ranged from 3.5 to 23 cm with a mean of 10.2 ± 4.3 cm. The liver cyst was localized to the right lobe in 32 (56%) patients, to the left lobe in 12 (21%) patients, while 12 (21%) patients had both liver lobes involved and 1 patient (2%) had an isolated caudate lobe cyst. The involved liver segments are shown in Table 1. The right posterior liver sector (segments 6 and 7) harbored 39 (39.8%) of the involved segments.

**Table 1:** Summary of the patient’s characteristics

Variable	Summary
Age (years)	37.2 ± 10.2
Gender (male:female)	38:19
Body mass index (kg/m^2^)	27 ± 4.3
Presentation	
Abdominal pain	33 (58%)
Cholangitis	6 (10.5%)
Abdominal swelling	6 (10.5%)
Incidentally found	12 (21%)
Characteristics of liver cyst	
Single: multiple	37: 20
Diameter (cm)	10.2 ± 4.3
Location right: left: bilobar: caudate	32: 12: 12: 1
Liver segments involved*	
1	1
2	8
3	12
4	10
5	13
6	20
7	19
8	15
Viable daughter cysts	37 (64.9%)*
Presence of biliary communication	28 (28.6%)*
Bilirubin (μmol/L)	20.0 ± 29.1
AST (μmol/L)	58.9 ± 94.8
ALT (μmol/L)	88.0 ± 167.6
ALP (μmol/L)	121.7 ± 93.1
American Society of Anesthesia classification (ASA)	
1	29
2	20
3	8
Procedure performed	
Partial pericystectomy	43
Total pericystectomy	8
Liver resection	6
Surgical approach	
Laparotomy	14
Laparoscopic	37
Robotic	6
Follow-up years	1.2 ± 1.4

*based on the total number of cysts

Out of the total cohort, six patients (10.5%) presented with hydatid disease affecting multiple organs, including the lung, spleen, and brain. Initially, the treatment prioritized the management of brain and lung cysts, followed by subsequent treatment of the liver cysts. Notably, patients with splenic cysts underwent laparoscopic management concurrently with the treatment of liver cysts. All patients received albendazole for at least 6 weeks before surgery except for two patients, one had a ruptured hydatid cyst with an urgent surgical intervention, and the other had an unusual severe cholestasis related to albendazole. ASA classification was 1, 2, and 3 in 29 (51%), 20 (35%), and 8 (14%) patients, respectively. In addition, three patients had endoscopic retrograde cholangiopancreatography (ECRP) for biliary drainage and stenting before surgery.

A total of 14 (24.6%) patients underwent conventional surgery (6 partial pericystectomy; 4 total pericystectomy; 4 liver resection), 37 (64.9%) patients had laparoscopic surgery (31 partial pericystectomy; 4 total pericystectomy; 2 liver resection) while 6 (10.5%) underwent robotic surgery (6 partial pericystectomy). Total pericystectomy and liver resection procedures were significantly higher in the laparotomy group compared to the minimally invasive group (*P* = 0.004). Other procedures, including cholecystectomy +/− common bile duct clearance, were done in 12 patients, and splenectomy in 3 patients. Conversion from laparoscopy to conventional surgery happened in 1 (2.7%) case due to bleeding, and none of the robotic cases.

Considering the total number of cysts, viable daughter cysts were encountered in 37 (64.9%) patients. Twenty-eight (28.6%) cysts were found to have bile inside or clear biliary communication, and surgical suturing of bile duct openings were done in 15 cases, 6 cases were managed with the conventional approach; 7 had laparoscopic intervention while 2 patients underwent robotic surgery.

There was no difference between the laparotomy and the minimal invasive groups regarding the patients’ characteristics, including age at the time of surgery, BMI, ASA status, cyst number, and size (Table 2). The laparotomy group tended to have more males and deranged liver function. However, it did not reach a statistical difference except for the liver transaminase ALT, which was significantly higher in the laparotomy group. Regarding the operative factors, more pericystectomy and liver resection procedures (57%) were done with laparotomy than with the minimally invasive approaches (14%) (*P* = 0.010). However, the operation time and blood loss were comparable between both groups.

**Table 2:** Comparing the laparotomy and the minimally invasive groups

Variables	Laparotomy	Minimally invasive	*P*-value
Age	30.8 ± 8.9	37 ± 10.6	0.753
Gender Male:Female	13:1	25:18	0.057
Body mass index (kg/m^2^)	26 ± 4.8	27.3 ± 4.2	0.361
Number of cysts Single: Multiple	8:6	19:14	0.707
Diameter (cm)	9.8 ± 5	10.4 ± 4.1	0.681
Bilirubin (μmol/L)	30.1 ± 40.1	15.6 ± 22.1	0.069
AST (μmol/L)	111 ± 162	44.5 ± 55.2	0.063
ALT (μmol/L)	202 ± 266	58 ± 103	0.018
ALP (μmol/L)	158 ± 117	115 ± 86	0.223
American Society of Anesthesia classification (ASA) 1 2 3	7 1 1	22 13 7	0.219
Procedure performed Partial pericystectomy Total pericystectomy Liver resection	6 4 4	37 4 2	0.004
Estimated blood loss (ml)	225 ± 127	142 ± 141	0.086
Operation time (min)	278 ± 97	226 ± 85	0.228
Complications Yes:No	7:7	12:31	0.314
Hospital stay (days)	7.4 ± 3.2	3.9 ± 1.6	<0.001

A total of 19 (33.3%) patients had perioperative complications. They were graded according to the Clavien-Dindo classification and classified as minor (grades 1 and 2) in 16 (84%) patients. Major complications were all grade 3 and reported in 3 patients as bile leak; two required percutaneous drainage, and the third had ERCP stenting (Table 3).

**Table 3:** Postoperative complications in different surgical approaches

Description	Laparotomy	Minimally invasive
Minor complications (grades 1 & 2)	6	10
Wound infection	2	1
Diarrhea and electrolyte disturbance	0	1
Leukocytosis and fever	1	2
Atelectasis	1	0
Maculopapular rash	1	0
Hypernatremia	0	1
Emphysema	0	1
Bile leakage (prolonged drainage throught the drain)	1	4
Major complications (grade 3)	1	2
Bile leakage (required percutaneous drainage)	0	2
Bile leakage (required ERCP stenting)	1	0

Overall, bile leak happened in 8 (14%) patients, and it resolved spontaneously in 5 patients, while 3 patients required percutaneous drainage or ERCP stenting (Table 3).

There was no significant difference (*P* = 0.314) between the two groups regarding the incidence of complications, as shown in Table 2. Conventional surgery, however, was associated with a significantly more extended hospital stay (*P* < 0.001). The cohort had no reported mortality and no cyst recurrence by the end of the follow-up period of this study.

## Discussion

The surgical management of hydatid liver cysts plays a crucial role in the overall treatment of this condition [9]. Over the past decade, many centers have increasingly adopted the laparoscopic approach, although patient selection criteria have varied. Initially, concerns arose regarding the risk of cyst content spillage during laparoscopy, which could lead to anaphylactic reactions, dissemination of the hydatidosis, and increased risk of recurrence. Consequently, patient selection was limited. However, indications for laparoscopy have since expanded, with fewer restrictions based on cyst size or type but with considerations regarding cyst location. Some centers still exclude cysts in liver segments 1 and 7, as well as cysts with biliary communication [13–15].

In this report, we present our experience with both conventional and minimally invasive approaches, including laparoscopic and robotic options, for the surgical management of hydatid liver cysts. Our analysis encompasses 98 hydatid liver cysts operated on in 57 patients, with 75% of cases performed using minimally invasive approaches. In addition, we extended the indications for using minimally invasive techniques to a broad spectrum of cases, regardless of cyst size, number, viability of daughter cysts, or the presence of biliary communication. Among our cohort, 65% of the cysts had viable daughter cysts, 20% were located in segments 1 and 7, and 29% had biliary communication.

Overall, the morbidity rate in our study was 33.3%, with no reported mortality among our patients and no cases of cyst recurrence during the follow-up period. Most complications were minor in nature, with bile leakage occurring in 14% of cases.

Bile leakage is recognized as the most common complication following surgical management of hydatid liver cysts, with reported incidences ranging from 6.2 to 36.2% in the literature [15–18]. In our series, the incidence of bile leakage was 14%, which is reasonable considering the inclusion of cysts with biliary communication. Identifying biliary communication can be challenging prior to or during surgery, and it may become evident later when bile leaks through the abdominal drain. Intraoperative measures such as applying clips or sutures can typically resolve the issue if recognized during surgery. The pressure inside a hydatid cyst, which ranges from 0 to 4.2 mm Hg on average, tends to counteract bile leakage, especially when employing minimally invasive approaches with intra-abdominal insufflation pressures of 10–12 mm Hg [15]. Bile leakage is typically only observed postoperatively through the abdominal drain. Therefore, it is advisable to routinely place an intra-abdominal drain near the remaining cyst cavity to facilitate drainage of the leaking bile duct until spontaneous closure occurs.

The literature reports postoperative morbidity rates between 8 and 25% in laparoscopic studies and 12 to 63% in conventional surgery series [14]. The rate of treatment-related deaths in laparoscopic series is nearly zero, whereas it ranges from 0 to 3% in open surgery series [13].

A recent study [4] involving 100 patients investigated two surgical approaches: laparoscopy and open surgery. Out of the total, 39 patients underwent laparoscopy while 61 patients opted for open surgery. In both groups, partial pericystectomy was performed on all patients. The study reported a total of 55 complications, with the most common being bile leakage at 16% and intra-abdominal fluid collection at 14%. Fortunately, there were no recorded instances of mortality, and the recurrence rate stood at 6%.

Another more extensive study examined 353 patients, with 60 of them undergoing laparoscopic surgery and the remaining 293 undergoing conventional surgery. Complications were observed in 13.3% of the laparoscopic group and 19.8% of the conventional group. However, no statistically significant difference was found between the two groups, and there were no reported cases of mortality in the study. The recurrence rate was 1.7% in both groups [5].

It is important to note that the latter study excluded 333 patients who were deemed unfit for general anesthesia or had specific conditions such as previous upper abdominal surgery, intrabiliary ruptured cyst, intraparenchymal located cyst, recurrent cyst, multiorgan cyst, cyst located in segments 1 and 7, and cyst larger than 15 cm in size [5].

In a recent review of 914 patients who underwent laparoscopic management of liver hydatid cysts, the overall morbidity rate was 15%, and the mortality rate was 0.2%. Conversion to open laparotomy occurred in 5% of cases, primarily due to limited access. Bile leakage was reported in 6.2% of cases, and the postoperative recurrence rate was 1.1% [14]. In our cohort, we observed no mortality among our patients, and the morbidity rate was 33.3%, with the majority of complications being minor, which is comparable to figures reported in the literature.

Treatment of hepatic hydatid cysts with chemotherapy alone, such as albendazole, is not as effective as a combined chemotherapy-drainage approach. Medical treatment often leads to clinical and radiographic improvements, such as cyst size reduction, membrane separation, or cyst calcification [19, 20] However, complete cure is generally achieved in less than half of the patients treated with antiparasitic monotherapy. Preoperative albendazole therapy has become routine in laparoscopic hydatid surgery, with a minimum duration of 4 weeks. This therapy effectively reduces cyst viability and intracystic pressure, minimizing the risk of spillage and implantation [19, 20].

The primary principles in the surgical management of hydatid liver disease involve parasite eradication, inactivation of the germinal membrane, prevention of cyst content dissemination into the peritoneal cavity, prevention of anaphylactic reactions, management of biliary communication, and minimal hepatic parenchymal damage [15]. The use of scolicidal agents during surgery has become an essential aspect of the surgical technique employed by surgeons worldwide. Various scolicidal agents, including warm water, chlorhexidine, hydrogen peroxide, ethyl alcohol, cetrimide, and hypertonic saline at different concentrations, have been tested. However, the effectiveness and safety of these agents remain uncertain.

The World Health Organization (WHO) has recommended a 20% hypertonic saline solution, which has demonstrated the ability to kill scolices within 10 min in laboratory tests (in vitro) [2, 21]. Unfortunately, this solution can potentially cause injury to the bile duct mucosa, leading to sclerosing cholangitis if there is communication with the biliary system. Moreover, its effects strongly depend on the concentration used. The impact of hypertonic saline is determined by its concentration rather than the duration of contact, and the degree of dilution inside the cyst is unpredictable [2, 21]. Therefore, we utilize different concentrations of hypertonic saline (5%, 10%, and 20% NaCl) based on the size of the cyst and the presence or absence of biliary communication. For instance, a 5% NaCl solution is typically employed for small cysts (<5 cm), while a 10% NaCl solution is used for cysts ranging from 5 to 10 cm. A 20% NaCl solution is reserved for cysts larger than 10 cm. As the cyst size increases, the injected fluid becomes more diluted. In cases where biliary communication is suspected, the injection of 20% hypertonic saline should be avoided.

Additionally, it is crucial to isolate the peritoneum by using packs soaked in either hypotonic or hypertonic saline. This measure aims to prevent the dissemination of cyst contents into the peritoneal cavity, thus reducing the risk of cyst recurrence. When dealing with a significant number of hydatid liver cysts, the utilization of hypotonic saline may be considered to minimize the chances of hyperosmolar peritoneal damage, systemic absorption, and the resulting hypernatremia [21]. Conversely, for cases characterized by a less severe hydatid liver disease, gauzes soaked in hypertonic saline are employed.

Ensuring reasonable access is crucial to achieve these objectives and should not be compromised by an inappropriate approach. Preoperative CT or MRI scans are essential for a detailed assessment of the disease and surgical planning, especially to evaluate the proximity of cysts to major vessels and exclude any exocytic brood capsules to avoid leaving viable parasites behind. Intraoperative ultrasound can be beneficial in these cases to ensure proper management and prevent the oversight of viable cysts.

Conventional surgery or laparotomy is a traditional surgical option for hepatic hydatid cysts. It offers the advantages of complete cyst removal and direct access to the cyst [13, 22]. However, the minimally invasive approaches provide potential benefits such as less pain, shorter hospital stay, and faster recovery time compared to conventional surgery [22]. The laparoscopic approach provides a magnified view of the cyst cavity, allowing for more precise suturing [22], while the da Vinci surgical system is a robotic-assisted surgical platform that enables surgeons to perform complex surgeries with increased precision and control, it is a relatively new and experimental technique, and its availability may be limited in some hospitals [6–8].

Our findings suggest that the minimally invasive approaches, including laparoscopy and robotics, can be safely and effectively employed in the surgical management of hydatid liver cysts. By expanding the indications for these techniques, we were able to successfully treat a wide range of cases with favorable outcomes. It is worth noting that our study did not observe any cyst recurrence, indicating the efficacy of the selected surgical approaches.

However, it is important to acknowledge that our study has some limitations, including its retrospective nature and the relatively small sample size. Further studies with larger patient populations and longer follow-up periods are needed to validate our findings and assess long-term outcomes, including cyst recurrence rates. Nonetheless, our results contribute to the growing body of evidence supporting the feasibility and effectiveness of minimally invasive approaches in the surgical management of hydatid liver cysts.

In conclusion, the use of laparoscopic and robotic techniques for treating hydatid liver cysts provides expanded treatment options, enabling successful outcomes regardless of cyst characteristics, with advantages including shorter hospital stays and potentially quicker recovery, making them valuable choices when combined with careful patient selection and appropriate surgical techniques.
